# Supplementary material for: Assessing Risks to Wildlife from Free-Roaming Hybrid Cats: The Proposed Introduction of Pet Savannah Cats to Australia as a Case Study
Source: Animals (Basel). 2019 Oct 14;9(10):795. doi: 10.3390/ani9100795 (PMC6826879; doi:10.3390/ani9100795)
Supplement: Supplementary file 1 [file animals-09-00795-s001.pdf]

Supplementary Table S1: Australian terrestrial mammal species assessed as more susceptible to predation from savannah cats than domestic cats.

We assume that any mammal species assessed as at risk of predation by the domestic cat will be at least as susceptible to predation by the savannah cat (SC). Only those mammal species assessed as more susceptible to predation by savannah cats than by domestic cats are listed here. The full list of all mammal species assessed is provided in Table S1 of Radford et al. 2018 [53]. Female body weight data are mostly from Van Dyck and Strahan 2008 [62], with data on species described since 2008 obtained from the primary sources.

Species	Common Name	Female Body Weight (g)	Conservation Status (IUCN) [46]	Susceptibility to Domestic Cat Predation *	Susceptibility to Savannah Cat Predation	Reason for Change
Ornithorhynchus anatinus	Platypus	600–1700	NT	Not susceptible	High	Occupies riparian areas, dense vegetation
Zaglossus bruijnii	Western long-beaked echidna	~5000	CR	Not susceptible	Low	Weight range places it at high potential susceptibility to SC predation, but spines provide some protection
Dasyurus maculatus	Spotted-tailed Quoll	900–2500	NT	Low	High	Partly arboreal, occupies dense wet forests
Sarcophilus harrisii	Tasmanian devil	5000–9000	EN	Not susceptible	Low	Weight range places it at high potential susceptibility to SC predation, but pugnacious behaviour reduces risk to low
Antechinus adustus	Rusty antechinus	18–35	LC	Not susceptible	Low	Occupies rainforest, some activity high above ground
Antechinus godmani	Atherton antechinus	42–78	NT	Not susceptible	Low	Occupies rainforest, some activity high above ground
Antechinus leo	Cinnamon antechinus	32–74	LC	Not susceptible	Low	Occupies rainforest, some activity high above ground
Antechinus mysticus	Buff-footed antechinus	18–34	LC	Not susceptible	Low	Occupies rainforest, some activity likely to be high above ground
Antechinus subtropicus	Subtropical antechinus	16–32	LC	Not susceptible	Low	Occupies rainforest, some activity high above ground
Myrmecobius fasciatus	Numbat	305–647	EN	Low	High	Weight range places it at high potential susceptibility to SC predation
Echymipera rufescens	Long-nosed echymipera	300–1200	LC	Low	High	Occupies rainforest, weight range places it at high potential susceptibility to SC predation
Isoodon macrourus	Northern brown bandicoot	500–1700	LC	Low	High	Often occupies dense vegetation, weight range places it at high potential susceptibility to SC predation
Isoodon obesulus	Southern brown bandicoot	400–1200	NT	Low	High	Often occupies dense vegetation, weight range

						places it at high potential susceptibility to SC predation
Isoodon fusciventer	Quenda	400–1100	LC	Low	High	Often occupies dense vegetation, weight range places it at high potential susceptibility to SC predation
Isoodon peninsulae	Cape York brown bandicoot	350–1050	LC	Low	High	Often occupies dense vegetation, weight range places it at high potential susceptibility to SC predation
Perameles pallescens	Northern long-nosed bandicoot	350–950	LC	Low	High	Often occupies dense vegetation, weight range places it at high potential susceptibility to SC predation
Phascolarctos cinereus	Koala	7000–11,000	VU	Not susceptible	Low	Weight range places it at some risk to SC predation, mostly active high above ground
Dactylopsila trivirgata	Striped possum	310–475	LC	Low	High	Occupies rainforest, often active near ground
Petaurus gracilis	Mahogany glider	310–454	EN	Not susceptible	Low	Usually active > 4 m above ground
Petropseudes dahli	Rock ringtail possum	1280–2000	LC	Low	High	Much activity in trees < 4 m above ground
Trichosurus caninus	Short-eared brushtail possum	2500–4500	LC	Low	High	Often active on ground in dense forest, weight range places it at high potential susceptibility to SC predation
Trichosurus cunninghami	Mountain brushtail possum	2600–4200	LC	Low	High	Often active on ground in dense forest, weight range places it at high potential susceptibility to SC predation
Trichosurus vulpecula	Common brushtail possum	1200–3500	LC	Low	High	Often active on ground in dense forest, weight range places it at high potential susceptibility to SC predation
Wyulda squamicaudata	Scaly-tailed possum	900–2000	NT	Low	High	Much activity in trees < 4 m above ground
Hypsiprymnodon moschatus	Musky rat-kangaroo	453–635	LC	Low	High	Occupies rainforest, weight range places it at high potential susceptibility to SC predation
Aepyprymnus rufescens	Rufous bettong	1300–3000	LC	Low	High	Part of range overlaps dense forest, weight range places it at high potential susceptibility to SC predation
Bettongia gaimardi	Eastern bettong	1200–2250	NT	Low	High	Part of range overlaps dense forest, weight range places it at high potential susceptibility to SC predation
Dendrolagus bennettianus	Bennett's tree-kangaroo	6300–10,600	NT	Not susceptible	Low	Occupies rainforest, mostly active > 4 m above ground

Dendrolagus lumholtzi	Lumholtzi's tree-kangaroo	5100–7750	NT	Not susceptible	Low	Occupies rainforest, mostly active > 4 m above ground
Lagorchestes conspicillatus	Spectacled hare-wallaby	1600–4750	LC	Low	High	Weight range places it at high potential susceptibility to SC predation
Notamacropus agilis	Agile wallaby	9000–15,000	LC	Not susceptible	Low	Weight range places it at low potential susceptibility to SC predation
Notamacropus dorsalis	Black-striped wallaby	5200–7600	LC	Not susceptible	High	Weight range places it at high potential susceptibility to SC predation
Notamacropus eugenii	Tammar wallaby	4000–6000	LC	Low	High	Weight range places it at high potential susceptibility to SC predation
Notamacropus irma	Western brush wallaby	7000–9000	LC	Low	High	Weight range places it at intermediate potential susceptibility to SC predation, high risk associated with occupation of wet flats and thickets
Notamacropus parma	Parma wallaby	3200–4800	NT	Not susceptible	High	Weight range places it at high potential susceptibility to SC predation
Notamacropus parryi	Whiptail wallaby	7000–15,000	LC	Not susceptible	Low	Weight range places it mostly at low potential susceptibility to SC predation
Notamacropus rufogriseus	Red-necked wallaby	11,000–15,500	LC	Not susceptible	Low	Weight range places it at low potential susceptibility to SC predation
Osphranter bernardus	Black wallaroo	13,000	NT	Not susceptible	Low	Weight range places it at low potential susceptibility to SC predation
Onychogalea fraenata	Bridled nailtail wallaby	4000–6000	EN	Low	High	Weight range places it at high potential susceptibility to SC predation
Onychogalea unguifera	Northern nailtail wallaby	4500–7000	LC	Low	High	Weight range places it at high potential susceptibility to SC predation
Petrogale herberti	Herbert's rock-wallaby	3700–4900	LC	Not susceptible	Low	Weight range places it at high potential susceptibility to SC predation, but its rock outcrop habitat likely reduces risk to low
Petrogale inornata	Unadorned rock-wallaby	3100–5000	LC	Not susceptible	Low	Weight range places it at high potential susceptibility to SC predation, but its rock outcrop habitat likely reduces risk to low
Petrogale	Proserpine rock-	3500–8100	EN	Not	Low	Weight range places it

persephone	wallaby			susceptible		at high potential susceptibility to SC predation, but its rock outcrop habitat likely reduces risk to low
Petrogale purpureicollis	Purple-necked rock-wallaby	2700–5700	NT	Not susceptible	Low	Weight range places it at high potential susceptibility to SC predation, but its rock outcrop habitat likely reduces risk to low
Thylogale billardierii	Tasmanian pademelon	2400–10,000	LC	Low	High	Occupies rainforest / wet forest with dense understorey, weight range places it mostly at high potential susceptibility to SC predation
Thylogale stigmatica	Red-legged pademelon	2500–4200	LC	Low	High	Occupies rainforest / wet forest with dense understorey, weight range places it at high potential susceptibility to SC predation
Thylogale thetis	Red-necked pademelon	1800–4300	LC	Low	High	Occupies rainforest / wet forest with dense understorey, weight range places it at high potential susceptibility to SC predation
Setonix brachyurus	Quokka	1600–3500	VU	Low	High	Mostly confined on mainland to dense vegetation in high rainfall areas, weight range places it at high potential susceptibility to SC predation
Wallabia bicolor	Swamp wallaby	10,300–15,400	LC	Not susceptible	Low	Weight range places it at low potential susceptibility to SC predation
Mastacomys fuscus	Broad-toothed rat	97–145	NT	Low	High	Occupies dense wet, often riparian habitats
Pseudomys nanus	Western chestnut mouse	18–56	LC	Low	High	Occupies varied habitats with dense understorey
Hydromys chrysogaster	Water-rat	340 – 992	LC	Low	High	Occupies riparian areas, often with dense vegetation
Xeromys myoides	Water mouse	32–64	VU	Low	High	Occupies coastal and riparian areas, often with dense vegetation
Melomys burtoni	Grassland melomys	26–97	LC	Low	High	Occupies dense vegetation in high rainfall coastal areas, much activity < 4 m above ground
Melomys capensis	Cape York melomys	45–96	LC	Not susceptible	High	Occupies rainforest and other wet forest, much activity < 4 m above ground
Melomys cervinipes	Fawn-footed melomys	45–120	LC	Not susceptible	High	Occupies dense vegetation in high rainfall coastal areas,

						much activity < 4 m above ground
Uromys caudimaculatus	Giant white-tailed rat	500–800	LC	Not susceptible	High	Occupies rainforest and other wet forest, much activity < 4 m above ground
Uromys hadrourus	Pygmy white-tailed rat	152–220	NT	Not susceptible	High	Occupies rainforest, much activity likely to be < 4 m above ground
Pogonomys sp.	Tree mouse	40–79	LC	Not susceptible	Low	Occupies rainforest, much activity likely to be > 4 m above ground
Rattus leucopus	Cape York rat	95–207	LC	Not susceptible	Low	Occupies rainforest, but much activity in logs, under litter or in burrows may reduce potential susceptibility to SC predation from high to low
Rattus tunneyi	Pale field-rat	42–85	LC	Low	High	Occupies tall grassland, usually near watercourses

* Radford et al. 2018 [53] assessed predator susceptibility to both the domestic cat and red fox together. We used evidence based on the relative impacts of foxes and cats in [53] to identify the susceptibility of each mammal species to domestic cat predation alone. For example, if a species was assessed as highly susceptible to predation by cats and foxes in [53], with the impact of foxes rated as being stronger than that of cats (cat < fox), we conservatively rated the susceptibility of that species to cats alone as low. If a species was assessed as being of high or low susceptibility to predation by cats and foxes in [53], with the impact of foxes being equal to that of cats (cat = fox), the susceptibility of that species to cats alone remained unchanged. IUCN conservation status: LC = Least Concern, NT = Near Threatened, VU = Vulnerable, EN = Endangered, CR = Critically Endangered.

References

62. Van Dyck, S.; Strahan, R. *The Mammals of Australia*, 4th edition; Reed New Holland: Sydney, Australia, 2008.
